# Neuropsychological outcome after cardiac arrest: a prospective case control sub-study of the Targeted hypothermia versus targeted normothermia after out-of-hospital cardiac arrest trial (TTM2)

**DOI:** 10.1186/s12872-020-01721-9

**Published:** 2020-10-07

**Authors:** Erik Blennow Nordström, Gisela Lilja, Susanna Vestberg, Susann Ullén, Hans Friberg, Niklas Nielsen, Katarina Heimburg, Lars Evald, Marco Mion, Magnus Segerström, Anders M. Grejs, Thomas Keeble, Hans Kirkegaard, Hanna Ljung, Sofia Rose, Matthew P. Wise, Christian Rylander, Johan Undén, Tobias Cronberg

**Affiliations:** 1 Lund University, Skane University Hospital, Center for Cardiac Arrest at Lund University, Neurology Research Unit, Department of Clinical Sciences Lund, Neurology, Remissgatan 4, 221 85 Lund, Sweden; 2 Lund University, Department of Psychology, Lund, Sweden; 3 Skane University Hospital, Clinical Studies Sweden – Forum South, Lund, Sweden; 4 Lund University, Skane University Hospital, Department of Clinical Sciences Lund, Intensive and Perioperative Care, Malmö, Sweden; 5 Lund University, Helsingborg Hospital, Department of Clinical Sciences Lund, Anesthesiology and Intensive Care, Lund, Sweden; 6 Hammel Neurorehabilitation and Research Centre, Hammel, Denmark; 7 Essex Cardiothoracic Centre, Basildon and Thurrock University Hospitals, Basildon, UK; 8 Sahlgrenska University Hospital, Department of Neurology and Department of Cardiology, Gothenburg, Sweden; 9 Aarhus University Hospital and Aarhus University, Research Centre for Emergency Medicine, Emergency Department and Department of Clinical Medicine, Aarhus, Denmark; 10 Department of Allied Health and Medicine, Anglia Ruskin School of Medicine, Chelmsford, UK; 11 Clinical Psychology, Cardiff and Vale University Health Board, NHS Wales, Cardiff, UK; 12 Cardiff University School of Medicine, Cardiff, UK; 13 Sahlgrenska Academy, University of Gothenburg, Institute of Clinical Sciences, Department of Anaesthesiology and Intensive Care Medicine, Gothenburg, Sweden; 14 Lund University, Skane University Hospital, Department of Clinical Sciences Malmö, Anaesthesiology and Intensive Care Medicine, Lund, Sweden

**Keywords:** Cardiac arrest, Cognitive dysfunction, Neuropsychological tests, Outcome, Cognitive screening

## Abstract

**Background:**

This study is designed to provide detailed knowledge on cognitive impairment after out-of-hospital cardiac arrest (OHCA) and its relation to associated factors, and to validate the neurocognitive screening of the Targeted Hypothermia versus Targeted Normothermia after Out-of-Hospital Cardiac Arrest trial (TTM2-trial), assessing effectiveness of targeted temperature management after OHCA.

**Methods:**

This longitudinal multi-center clinical study is a sub-study of the TTM2-trial, in which a comprehensive neuropsychological examination is performed in addition to the main TTM2-trial neurocognitive screening. Approximately 7 and 24 months after OHCA, survivors at selected study sites are invited to a standardized assessment, including performance-based tests of cognition and questionnaires of emotional problems, fatigue, executive function and insomnia. At 1:1 ratio, a matched control group from a cohort of acute myocardial infarction (MI) patients is recruited to perform the same assessment. We aim to include 100 patients per group. Potential differences between the OHCA patients and the MI controls at 7 and 24 months will be analyzed with a linear regression, using composite *z*-scores per cognitive domain (verbal, visual/constructive, working memory, episodic memory, processing speed, executive functions) as primary outcome measures. Results from OHCA survivors on the main TTM2-trial neurocognitive screening battery will be compared with neuropsychological test results at 7 months, using sensitivity and specificity analyses.

**Discussion:**

In this study we collect detailed information on cognitive impairment after OHCA and compare this to a control group of patients with acute MI. The validation of the TTM2 neurocognitive screening battery could justify its inclusion in routine follow-up. Our results may have a potential to impact on the design of future follow-up strategies and interventions after OHCA.

**Trial registration:**

ClinicalTrials.gov, NCT03543371. Registered 1 June 2018

## Background

Cardiac arrest is the abrupt loss of cardiac function and circulation, followed by loss of consciousness. Acute myocardial infarction (MI) is the result of acute coronary ischemia and necrosis. Many out-of-hospital cardiac arrests (OHCA) are caused by ventricular arrhythmias complicating acute MI [1]. However, there are many other cardiac and non-cardiac causes of cardiac arrest. The main difference in outcome between acute MI and OHCA is related to the circulatory standstill and subsequent brain injury in OHCA.

Since mortality and morbidity after successful resuscitation from OHCA is mostly due to brain injury [2], neurologic function is an important outcome measure. More than 90% of OHCA survivors have been found to have a favorable neurological outcome [3], when assessed with the commonly used clinician-based outcome scales such as the Cerebral Performance Categories Scale [4] or the modified Rankin Scale [5]. These scales may however miss important information regarding milder impact on cognitive and emotional functions [6].

From a neuropsychological point of view, cognitive impairment and emotional problems are common post-arrest. Cognitive impairment has been observed in about half of OHCA survivors [7–9]. A sub-study of the Target Temperature Management trial reported impairments in memory, executive functions or processing speed [10]. These cognitive domains are often impaired post-arrest, but also the most commonly assessed [11]. Others have reported that language and visual/constructive functions may be affected as well [12–14]. Brain damage is often diffuse with multiple atrophic areas due to transient global cerebral ischemia [15, 16].

The exact causes of cognitive impairment post-arrest are not yet known. An earlier study from our group found only minor differences between cognition in OHCA survivors and a gender and age-matched group with acute ST-elevation myocardial infarction (STEMI) without OHCA after 6 months follow-up [10]. This suggests that cardiovascular risk factors such as vascular degeneration, diabetes, hypertension, smoking, dyslipidemia, and physical inactivity may contribute to the cognitive impairment in both patient groups. However, these findings need to be corroborated with more sensitive tests. It is also unclear if the minor between-group cognitive differences that were detected persist over a longer time frame, or when using more detailed methods. In this study, we will again use a group of matched acute MI patients as controls. This choice of control group will enable us to separate the effects of the cardiovascular risk factors from cognitive impairment due to hypoxic-ischemic brain injury caused by the OHCA.

The greatest recovery of cognitive function is usually seen in the first 3 months post-arrest, but evidence from several small studies indicates continued improvement in some functions during at least the first year [17, 18]. There is a lack of follow-up with neuropsychological instruments examining several cognitive domains. Earlier studies on cognition after OHCA have either included a smaller number of participants (less than 50), or investigated more patients (100–300) with elementary cognitive assessments or fewer cognitive domains [10, 17, 19].

Cognitive impairment post-arrest correlates with emotional problems [20]. Symptoms of depression have been found in 14 to 45% of all survivors, anxiety in 13 to 61%, and posttraumatic stress in 19 to 27% [21]. Fatigue is closely related to cognitive and emotional function and has been reported in 69% of survivors [22]. The full relationship between fatigue and cognitive impairment, emotional problems and insomnia has yet not been investigated in OHCA survivors.

A cognitive and emotional screening post-arrest is recommended [23, 24] and could identify patients with potential sequelae, thus offering support, rehabilitation and if needed a more extensive assessment. Ideally, a follow-up screening battery should have a low threshold and high sensitivity so that even a slight impairment can be detected for further interventions, such as a detailed neuropsychological evaluation. The Targeted Hypothermia versus Targeted Normothermia after Out-of-Hospital Cardiac Arrest trial (TTM2-trial) is an international, multicenter, randomized trial in which a target temperature of 33 °C after OHCA will be compared with a strategy to maintain normothermia and early treatment of fever (≥37.8 °C) during the first 40 h after arrest [25]. The trial will randomize 1900 patients and includes a brief neurocognitive screening of survivors at 6 and 24 months.

This sub-study of the TTM2-trial aims, using a comprehensive neuropsychological test battery, to provide detailed and longitudinal information on cognitive impairment in OHCA survivors in comparison to a control group with acute MI, and the associated factors emotional problems, fatigue, insomnia, and vascular cognitive decline. In addition, this study aims to validate the main TTM2-trial neurocognitive screening, which could be suitable to include in routine follow-up if showing sound psychometric properties.

### Hypotheses

- OHCA survivors will perform significantly worse on neuropsychological tests of cognition compared to a matched cohort of acute MI patients without cardiac arrest.
- The neurocognitive screening used in the main TTM2-trial will with high sensitivity and adequate specificity identify OHCA survivors with cognitive impairment at 7 months post-arrest.

## Methods

### Study design

This sub-study of the TTM2-trial is a prospective non-intervention multi-center clinical study, in which selected sites participate.

### Participants

Inclusion and exclusion criteria [25] are listed in Table 1.

**Table 1:** Inclusion and exclusion criteria for the TTM2 neuropsychological sub-study

**Inclusion criteria**		**Main TTM2-trial**	**TTM2 Neuropsychological sub-study OHCA patients**	**TTM2 Neuropsychological sub-study MI patients**
	OHCA of a presumed cardiac or unknown cause	X	X	
	Sustained ROSC during intensive care – defined as 20 min with signs of circulation without the need for chest compressions	X	X	
	Unconsciousness – defined as not being able to obey verbal commands (FOUR-score motor response of < 4) and no verbal response to pain after sustained ROSC	X	X	
	Inclusion within 180 min of ROSC	X	X	
	During intensive care at admission – eligible for intensive care without restrictions or limitations	X	X	
	Acute MI with performed coronary angiography			X
**Exclusion criteria**	Temperature on admission < 30 °C	X	X	
	On ECMO prior to return of spontaneous circulation	X	X	
	Obvious or suspected pregnancy	X	X	
	Intracranial bleeding	X	X	
	Severe COPD with long-term home oxygen therapy	X	X	
	Age < 18	X	X	X
	Age ≥ 80		X	X
	Clinical dementia diagnosis before the event		X	X
	Inability to speak the local language well enough to complete the assessment without assistance from an interpreter		X	X
	Inability to meet for a face-to-face examination		X	X
	Active drug abuse		X	X
	Clinical Frailty Scale Index ≥8, indicating very severe frailty [26]		X	X
	Cardiac arrest before or in connection with MI			X

*OHCA* out-of-hospital cardiac arrest; *MI* myocardial infarction; *ROSC* return of spontaneous circulation; *ECMO* extracorporeal membrane oxygenation; *COPD* chronic obstructive pulmonary disease

A control group from a cohort of patients with confirmed and treated acute MI who underwent coronary angiography but without occurrence of cardiac arrest will be recruited at 1:1 ratio. Patients with STEMI and Non-STEMI are eligible as controls. Exclusion criteria for the MI-cohort are the same as for OHCA survivors.

No sample size calculation was performed due to a lack of earlier studies comparing OHCA and MI survivors by using the tests in this study. Instead, our aim is to include a convenience sample of 100 patients per group at 7 months after the event.

### Ethical approval, informed consent and trial registration

Ethical applications for the TTM2-trial and this sub-study were approved by all participating countries. All eligible patients at participating sites will receive written and oral information and be asked to take part in the neuropsychological sub-study. Patients will be asked to sign a written informed consent. The TTM2-trial and this sub-study are registered at ClinicalTrials.gov (NCT02908308 and NCT03543371).

### Procedure

The main TTM2-trial follow-up at 6 months includes a neurocognitive screening [27], which consists of the performance-based instruments Montreal Cognitive Assessment (MoCA) [28] and Symbol Digit Modalities Test (SDMT) [29], the patient-reported Two Simple Questions (TSQ) [30, 31] and the observer-report Informant Questionnaire on Cognitive Decline in the Elderly-Cardiac Arrest (IQCODE-CA) [32, 33].

During the 6-month follow-up of the TTM2-trial, OHCA survivors at participating sites will be invited to partake in this sub-study. At a separate face-to-face appointment at 7 months (preferably within 28 days after regular follow-up) specific to this sub-study, a standardized neuropsychological battery will be administered. The battery comprises performance-based tests of major cognitive domains; verbal, visual/constructive, working memory, episodic memory, processing speed, and executive functions. Instruments will be administered according to guidelines in each manual, including criteria for discontinuing single instruments. The examination will be completed in one single session per follow-up, in a predefined order, and requires approximately 65–85 min with small breaks allowed. Neuropsychologists, psychologists, assistant clinical psychologists, and psychology students (under supervision of a psychologist) will administer the tests after participating in an introductory/training session. Patients and informants are asked to fill out four questionnaires of emotional problems, fatigue, executive dysfunction and insomnia prior to, or after the visit. Three of the questionnaires are self-report measures and will be returned by the patient. The fourth questionnaire is to be completed by an informant, i.e. a relative or close friend. This questionnaire could be returned in a pre-paid envelope by regular mail, in order to maintain the privacy of the informant.

The same neuropsychological test battery will be repeated 24 months post-arrest to investigate to what extent the long-term cognitive outcome of the survivors is accurately predicted by the 7 months examination.

**Fig. 1:**
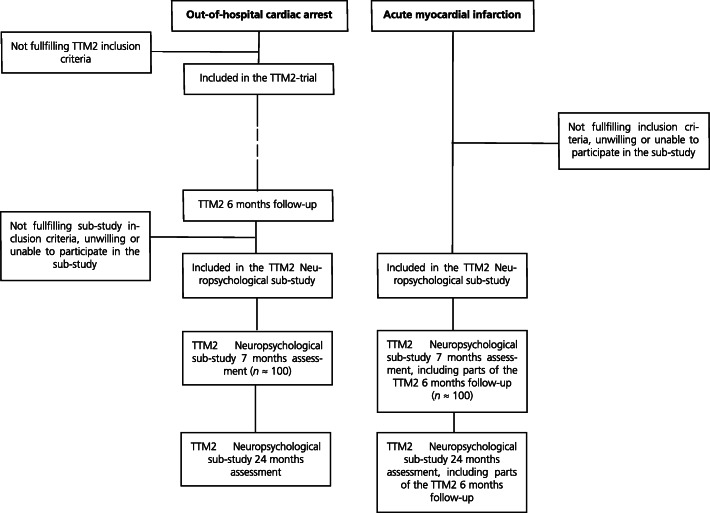
Flowchart of TTM2 neuropsychological sub-study inclusion. TTM2, Targeted Hypothermia versus Targeted Normothermia after Out-of-Hospital Cardiac Arrest trial

For each participating OHCA-survivor a MI control patient will be recruited per mail and telephone and assessed at one participating site per country (Fig. 1). The MI controls are matched according to the following matching criteria in a descending order: 1) date of cardiac event (all MI within a time frame of ±4 weeks of the CA are accepted); 2) sex; 3) age (best match). For MI control patients, descriptive background information and information on cardiovascular risk factors will be collected through objective measures and structured interview, while the corresponding information for the OHCA survivors will be collected during the regular TTM2 follow-up when applicable (Table 2).

**Table 2:** Descriptive background data used in the TTM2 neuropsychological sub-study

Variable	
Age	
Level of education	
Living situation	
Working status before and post event	
Results of the MoCA	
Results of the SDMT	
Results of the TSQ	
Results of the IQCODE-CA	
Current smoking	
Current diabetes	
Total cholesterol	
HDL cholesterol	
Systolic blood pressure	
HBA1c	
Length	
Weight	
Any current pharmaceutical treatment for hypercholesterolemia	
Any current pharmaceutical treatment for diabetes	
Any current pharmaceutical treatment for high blood pressure	
Any current pharmaceutical treatment for agitation/anxiety^#^	
Any current pharmaceutical treatment for depression^#^	
Any current pharmaceutical treatment for insomnia^#^	

The “^#^” indicates the background data which are not available through the main TTM2-trial follow-up and exclusively collected for this sub-study

*MoCA* Montreal Cognitive Assessment; *SDMT* Symbol Digit Modalities Test; *TSQ* Two Simple Questions; *IQCODE-CA* Informant Questionnaire on Cognitive Decline in the Elderly-Cardiac Arrest; *HDL* high-density lipoprotein

The meeting is preferably performed in a clinical setting, but alternative settings could be considered such as visiting the patient’s place of residence.

### Study timeline

The TTM2-trial started on November 18, 2017. The current sub-study started on July 13, 2018. Sites in Sweden, the United Kingdom and Denmark are recruiting. We estimate that the last patients are recruited in 2020, with the final longitudinal follow-up completed 2022.

### Study organization

This is an academically initiated study led by researchers at Center for Cardiac Arrest at Lund University.

The study is performed in cooperation with the TTM2 sub-study of Physical Activity After Cardiac Arrest (NCT03543332). If patients choose to participate in this parallel sub-study, the appointments are merged.

### Instruments

An overview of the neuropsychological test battery, including reliability coefficients, is found in Table 3, with scores used for computing composite *z-*scores per cognitive domain appearing in bold. The questionnaires are presented in Table 4.

**Table 3:** Tests and scores used in the TTM2 neuropsychological sub-study, grouped by cognitive domain

Cognitive domain	Cognitive functions	Test	Scores	Test-retest reliability	Administration order
Verbal	• Verbal comprehension • Concept verbalization • Semantic memory retrieval	WAIS-IV Vocabulary	**Total score**	High (.89)	5
	• Phonemic fluency • Semantic memory retrieval • Working memory capacity • Processing speed	D-KEFS Verbal Fluency	**Letter fluency score, category fluency score**, category switching score, category switching accuracy, first interval, second interval, third interval, fourth interval, set-loss errors, repetition errors	Letter fluency: High (.80) Category fluency: Adequate (.79)	13
Visual/constructive	• Visuospatial organization • Visuomotor speed	WAIS-IV Block Design	**Total score**, no time bonus score	High (.80)	2
	• Non-verbal abstract reasoning • Perceptual organization	WAIS-IV Matrix Reasoning	**Total score**	Adequate (.74)	4
Working memory	• Verbal short-term working memory • Aspects of auditory attentional capacity	WAIS-IV Digit Span	**Total score**, digit span forwards score, digit span forwards longest, digit span backwards score, digit span backwards longest, digit span sequencing score, digit span sequencing longest	Entire subtest: High (.83) Sub-conditions: Adequate (.71–.77)	10
	• Spatial working memory	WMS-III Spatial Span	**Total score**, spatial span forward, spatial span backward	Adequate (.71)	3
Episodic memory	• Verbal episodic list memory • Attention • Verbal recognition memory	RAVLT	**Total recall, delayed recall**, delayed recognition, trial 1, trial 2, trial 3, trial 4, trial 5, trial 6, trial 7	Total and delayed recall: Adequate to high (.74–.88) Other trials: Lower [34]	7, 12
	• Verbal episodic memory of prose passages • Verbal learning	WMS-III Logical Memory	**Total score I, total score II**	Adequate (.76–.77)	1, 6
	• Visual episodic memory • Visual learning • Visual recognition memory	BVMT-R	**Total recall, delayed recall**, recognition discrimination index, trial 1, trial 2, trial 3, learning, percent retained, recognition hits, recognition false alarms	Total recall: High (.80) Other trials: Lower	11, 16
Processing speed	• Visuomotor processing speed • Attention	TMT A	**Score**	Varies but mostly adequate, negligible practice effects over longer retest intervals [35]	8
	• Word naming speed • Color naming speed	D-KEFS Color Word Interference Test	**Color naming, word reading**	Color naming: Adequate (.76) Word reading: Marginal (.62)	14
Executive functions	• Cognitive flexibility • Working memory • Visuomotor processing speed	TMT B	**Score**	As TMT A above	9
	• Inhibition of a dominant and automatic verbal response • Sustained selective attention	D-KEFS Color Word Interference Test	**Inhibition, inhibition total errors**	Adequate (.76)	15

*WAIS-IV* Wechsler Adult Intelligence Scale – Fourth Edition; *D-KEFS* Delis-Kaplan Executive Function System; *WMS-III* Wechsler Memory Scale – Third Edition; *RAVLT* Rey Auditory Verbal Learning Test; *BVMT-R* Brief Visuospatial Memory Test-Revised; *TMT* Trail Making Test

*Note*: Scores used for computing composite *z-*scores per cognitive domain appear in bold

**Table 4:** Questionnaires filled out by the patient or the informant

Focus	Questionnaire	Respondent	Number of questions	Test-retest reliability
Depression and anxiety symptom screening	HADS	Patient	14	In an acute myocardial infarction sample: Acceptable [36]
Dysexecutive function	BADS DEX Self	Patient	20	In an acquired brain injury sample: High (.88) and marginal for informants (.60) [37]
Dysexecutive function	BADS DEX Other	Informant	20	In an acquired brain injury sample: Marginal for informants (.60) [37]
Fatigue; general fatigue, physical fatigue, reduced activity, reduced motivation and mental fatigue	MFI-20	Patient	20	Adequate (.76), somewhat lower for the subscale scores [38]
Insomnia screening	MISS	Patient	3	Unknown

*HADS* Hospital Anxiety and Depression Scale; *BADS DEX* Behavioral Assessment of the Dysexecutive Syndrome Dysexecutive Questionnaire; *MFI-20* Multidimensional Fatigue Inventory; *MISS* Minimal Insomnia Symptom Scale

### Verbal domain

The Vocabulary subtest from the Wechsler Adult Intelligence Scale – Fourth Edition (WAIS-IV) [39] assesses knowledge of words, abilities to verbalize concepts and retrieve information from the semantic memory.

The Letter Fluency condition of the Verbal Fluency subtest from the Delis-Kaplan Executive Function System (D-KEFS) [40] assesses oral productions of words beginning with a specified letter. The Category Fluency condition assesses oral production of words within a designated category. Letter Fluency is proposed to put greater emphasis on executive aspects such as attention allocation and strategic organization than Category Fluency [41]. However, both phonemic and semantic fluency has been reported to load exclusively on a language factor [42].

### Visual/constructive domain

The Block Design subtest from the WAIS-IV [39] assesses visuospatial organization. Bonus points are given for a quick, error-free performance. It is relatively free from cultural and educational bias, and is generally sensitive to any kind of cerebral dysfunction [43].

The Matrix Reasoning subtest from the WAIS-IV [39] is a test of non-verbal abstract reasoning and perceptual organization.

### Working memory domain

The Digits Forward condition of the Digit Span subtest from the WAIS-IV [39] emphasizes attention efficiency and auditory short-term memory. Digits Backwards emphasizes aspects of working memory, as does Digit Sequencing. The latter conditions are more difficult and rather sensitive to brain injury or deterioration [44].

The Spatial Span subtest from the Wechsler Memory Scale – Third Edition (WMS-III) [45] tests visuospatial working memory. The Backward condition is more sensitive to cognitive impairment than the Forward condition, requiring more complex working memory and visual manipulation skills [46].

### Episodic memory domain

The Rey Auditory Verbal Learning Test (RAVLT) [47] is a test of verbal learning and verbal episodic memory, by immediate recall in five trials, delayed recall and recognition of a word list.

In the Logical Memory subtest from the WMS-III [45], the subject is asked to recall two stories immediately and delayed.

The Brief Visuospatial Memory Test-Revised (BVMT-R) [48] measures visual learning and visual episodic memory by immediate recall in multiple trials, delayed recall and recognition. There are six different test forms of the BVMT-R with equivalent psychometric properties.

### Processing speed domain

The Trail Making Test (TMT) [49, 50] subtask A is a test of visual scanning with a motoric component. The TMT is vulnerable to brain dysfunction in general [43].

The Color-Word Interference Test (CWIT) from the D-KEFS [40] originates from the classic Stroop paradigm [51]. In its first two conditions, the CWIT measures processing speed.

### Executive functions domain

The TMT subtask B measures executive aspects such as cognitive flexibility in addition to processing speed [49, 50].

The third condition of the CWIT measures executive functions such as inhibition of a dominant and automatic verbal response, and sustained selective attention [40].

### Questionnaires

The Hospital Anxiety and Depression Scale (HADS) is a screening for symptoms of anxiety and depression by self-report [52]. The questionnaire consists of seven items for an anxiety subscale and seven items for a depression subscale. Each item is scored on a four-point scale, with responses totaled to obtain two subscale scores. A cut-score of ≥8 per subscale is considered to have a good balance between sensitivity and specificity, with most studies confirming the two-factor structure [53].

The Dysexecutive Questionnaire (DEX) from the Behavioral Assessment of the Dysexecutive Syndrome (BADS) [54] is designed to measure dysexecutive symptoms in everyday situations such as planning problems, distractibility and lack of insight. Items are rated on a five-point scale from zero to four, with higher scores representing greater problem severity. One version of the questionnaire should be completed by the subject (DEX-Self) and another version by an informant (DEX-Other). Discrepancies between the DEX-Self and DEX-Other are indicative of impaired insight [54]. A recent study using exploratory factor analysis on a large clinical and non-clinical sample resulted in confirmation of a one-factor solution for the DEX [55].

The Multidimensional Fatigue Inventory (MFI-20) measures fatigue in the past week in five dimensions: General fatigue, Physical fatigue, Reduced activity, Reduced motivation, and Mental fatigue [56, 57]. Items are rated on a five-point Likert scale, with an optional total score obtained by adding up the subscale scores. Higher scores indicate a higher degree of fatigue.

The Minimal Insomnia Symptom Scale (MISS) is an insomnia-screening questionnaire [58]. Items are rated on a five-point scale, yielding a total score ranging from 0 to 12 with higher scores indicating more insomnia. Rasch modeling has shown that a cut-score of ≥6 seems to be suited for adults and elderly when identifying presence of insomnia, although clinical criteria recently has been revised [59].

### Statistical analyses

Raw scores from all neuropsychological instruments will be converted to standardized *z*-scores in accordance with normative sample data (based on age and, when applicable, education) for each test. The *z*-scores will be adjusted for level of education and sex as covariates, when not already adjusted for in the *z*-score transformation. Test data will be grouped based on the six main cognitive domains measured by the tests (verbal, visual/constructive, working memory, episodic memory, processing speed, executive function). A composite *z*-score will be computed for each cognitive domain in accordance with Table 3, which also indicates the test scores used for composite *z-*scores computation. Parametric tests will be used for all analyses because of the *z*-score standardization and, in accordance with the central limit theorem, a large number of participants per group. A *p* of <.05 is considered significant for all analyses.

The first hypothesis will be analyzed with a linear regression adjusting for level of education and sex when examining the outcome at 7 months. These results will be reported separately. The analyses will be repeated at 24 months. Composite *z*-scores per cognitive domain are used as primary outcome measures.

The second hypothesis will be analyzed with a comparison of the OHCA survivors’ results on the neurocognitive screening and the detailed neuropsychological test battery at 7 months’ time. This will be performed by a sensitivity and specificity analysis emphasizing high sensitivity and adequate specificity, including true and false positive rates. Here, patients will be considered to have a cognitive impairment if they meet any of the following criteria on the neuropsychological tests: 1) an impaired composite *z*-score defined as *z* ≤ − 1.65 in at least one cognitive domain; 2) *z* ≤ − 1.65 in at least two scores used for cognitive domain calculation as per Table 3, independent of their domain classification. The proportion of OHCA survivors with impairments on neuropsychological tests will be compared with the proportion of OHCA survivors with indicated cognitive impairment on the neurocognitive screening, by: 1) performance-based instruments (MoCA, SDMT); 2) self and informant-report instruments (TSQ question 2, IQCODE-CA); 3) all four instruments together. As high sensitivity is of importance for a screening, just one score below cut-off on any screening instrument will indicate possible cognitive impairment. Cut-scores for indicated impairment will be < 26 on the MoCA, *z* ≤ − 1 on the SDMT, ≥3.08 on the IQCODE-CA and the answer “no” to question 2 at the TSQ.

An explorative approach will be used to analyze changes in OHCA survivors’ cognition and emotional problems, fatigue, insomnia, and cardiovascular risk factors from 7 to 24 months.

Missing data will be described and categorized as missing at random and missing not at random.

## Discussion

This neuropsychological sub-study of the TTM2-trial is a prospective, longitudinal multi-center clinical study. It is designed to provide detailed knowledge on cognition after cardiac arrest and its relation to emotional problems, fatigue, insomnia, and cardiovascular risk factors. This will provide valuable information as the number of survivors with cognitive impairment is still unclear, and mainly based on results using relatively crude instruments. Survivors will be followed longitudinally until 24 months post-arrest, which has not been done in other studies to date with a corresponding level of detail.

Another aim of this study is to compare patients with and without OHCA. Rather than a group of healthy controls, we have chosen to include a MI cohort as controls, similar to an earlier study from our group [10]. The detailed neuropsychological profile could be of use when tailoring future domain specific rehabilitation interventions for both patient groups. The matched MI controls in this study are recruited in the same country as the OHCA survivors in an effort to bridge any potential differences in demographics and rehabilitation between countries.

The neuropsychological test battery has been designed to provide detailed information about the cognitive function of the patients, while still being feasible to perform in one single session. A total of six cognitive domains are assessed, with a minimum of two tests per cognitive domain to reduce measurement errors. The tests comprising one cognitive domain are intended to measure the same overall cognitive function but different sub-components thereof, which enable analyses of specific test results and functions. The instruments were selected to represent a range of cognitive, behavioral and emotional functions. They are used in clinical practice as well as in research, and the majority of the instruments have previously been used in OHCA studies. All of the neuropsychological tests and most of the questionnaires have sound psychometric norms available.

The results of this study will have potential impact on the planning of future interventions. By validating the commonly used instruments in the neurocognitive screening of the TTM2-trial with neuropsychological instruments, the screening could be sufficient to serve as a model for future OHCA follow-up. Screening instruments with good validity enables the identification of patients in need of more detailed neuropsychological examination, follow-up and rehabilitation.

In addition to the follow-up assessments, the TTM2 population will be well investigated in the acute phase. This includes measures of electroencephalography, cerebral imaging, clinical neurological examination, and biomarkers in serum. Adding the detailed neuropsychological assessment of this study to the analyses, we will be able to investigate the importance of the acute brain injury and the chronic degenerative process post-arrest for neuropsychological functioning over time.

We have chosen to perform the sub-study specific neuropsychological assessment as a separate appointment, rather than at the same time as the regular TTM2 follow-up. This is due to fatigue being a common symptom after OHCA and acquired brain injury in general. Patient weariness and reduced vigilance thus could have implications for neuropsychological test performance [60]. Potential participants, especially those living far away from the study sites, could be more unwilling to partake as a result of this study design. To improve the inclusion ratio and the generalizability of the results, patients are offered a travel reimbursement or that the examination is performed in e.g. the patient’s place of residence.

There is no consensus on how to report cognitive decline in neuropsychological studies. Educational duration is positively correlated with premorbid intellectual capacity [61]. Striving for high specificity, especially in the neuropsychological test battery, we adjust for level of education in our main outcome measures. When comparing the results on the neurocognitive screening with the detailed neuropsychological test battery, the chosen cut-score of *z* ≤ − 1.65 was inspired by a similar OHCA study [12].

We have refrained from pre-specified comparisons of the neuropsychological function between the 33 °C and normothermia treatment arms. Differences in outcome between the temperature groups will primarily be investigated within the main TTM2-trial with a larger number of participants [27].

As for limitations, the TTM2-trial includes OHCA patients with presumed cardiac cause, however a substantial portion of the patients may have a non-cardiac cause. Although an exclusion of such patients would be possible in this sub-study, recruiting at 7 months post-arrest, we have refrained from this possibility because one of our aims is to validate the main TTM2-trial neurocognitive screening. Since all our MI controls will have a coronary disease, this will cause a discrepancy in our background variables. Still, a similar matching was performed in another study, with relatively equivalent cardiovascular risk factors between groups [10]. Another limitation is possible selection bias, i.e. that those with excellent recovery may be more likely to say yes to being part of the study, skewing the data in a positive way. These tendencies have been observed in other studies [10, 12, 17]. On the other hand, we have information from the main TTM2-trial on the patients declining to participate in this sub-study, facilitating analyses of missing data. Furthermore, the number of studies comparing neuropsychological outcome after OHCA and MI are few, and the tests used in this study are more sensitive than in most earlier studies. Thereby no sample size calculation could be performed for this study; however, the detailed and sensitive information will enable us to explore hypotheses generating trends in a large sample of cardiac arrest survivors and controls.

## Data Availability

The data that will support the findings of this study are available from the TTM2 steering group, but restrictions apply to the availability of these data, and so are not publicly available. Data are however available from the authors upon reasonable request and with permission of the TTM2 steering group.

## Abbreviations

TTM2-trial: Targeted hypothermia versus targeted normothermia after out-of-hospital cardiac arrest trial
OHCA: Out-of-hospital cardiac arrest
STEMI: ST-elevation myocardial infarction
MI: Myocardial infarction
MoCA: Montreal cognitive assessment
SDMT: Symbol digit modalities test
TSQ: Two simple questions
IQCODE-CA: Informant questionnaire on cognitive decline in the elderly-cardiac arrest
WAIS-IV: Wechsler adult intelligence scale – fourth edition
D-KEFS: Delis-Kaplan executive function system
WMS-III: Wechsler memory scale – third edition
RAVLT: Rey auditory verbal learning test
BVMT-R: Brief Visuospatial memory test-revised
TMT: Trail making test
CWIT: Color-word interference test
HADS: Hospital anxiety and depression scale
DEX: Dysexecutive questionnaire
BADS: Behavioral assessment of the Dysexecutive syndrome
MFI-20: Multidimensional fatigue inventory
MISS: Minimal insomnia symptom scale
